# Nurturing hope in Rwandan healthcare settings: Exploring factors that influence hope among healthcare providers, pregnant women, and mothers with children under five years

**DOI:** 10.1371/journal.pgph.0005095

**Published:** 2025-08-29

**Authors:** Angele Bienvenue Ishimwe, Annick Gloria Uwitonze, Sarah Mullane, Valens Hafashimana, Diana Nambatya Nsubuga, Sandra Isano, Diana Rujema, Talia Gramiccia, Wendy Leonard

**Affiliations:** 1 TIP Global Health, Kigali, Rwanda; 2 Health Tech & Innovation, Johnson & Johnson, London, United Kingdom; 3 Department of Global Community Based Education, University of Global Health Equity, Kigali, Rwanda; 4 UNICEF, Lebanon; Aga Khan University, PAKISTAN

## Abstract

According to the World Health Organization, inadequate antenatal care increases risks for both mothers and children, many of which can be prevented through regular screening, timely treatment, and adoption of healthy behaviors. Evidence suggests that healthcare recipients (HCRs) who receive quality care tend to be more hopeful and engaged in their care, while hope among healthcare providers (HCPs) can reduce burnout and enhance their ability to deliver quality services. This study explores the role of hope in healthcare delivery among HCRs and HCPs in rural Northern Rwanda, focusing on three key dimensions of hope: interconnectedness, readiness for change, and future orientation. Using a qualitative design, the research draws on focus group discussions and key informant interviews. Thematic analysis was conducted to identify key factors influencing hope and to generate recommendations for embedding hope in healthcare at individual, interpersonal, and health system levels. The analysis mapped a quadruple of areas serving as both facilitators and barriers of hope; relationships, care experiences, continuous learning, and working conditions. Respectful communication by HCPs fostered trust and positively influenced HCRs’ engagement. Faith, learning opportunities, and positive health system encounters supported future orientation and confidence. While HCPs showed strong commitment to care, their motivation was often challenged by resource constraints and poor working environments. The study highlights the importance of aligning quality care strategies with factors that promote hope. Strengthening relational care, promoting continuous learning, and improving work conditions can foster a more hopeful and resilient health system. Prioritizing such approaches can enhance both patient engagement and provider well-being across primary care settings.

## Introduction

Sustainable Development Goal (SDG) 3, established by the United Nations in 2015, aims to end preventable maternal, newborn, and child deaths by 2030. Across the globe, maternal deaths remain a challenge and a symbol of gross inequity. Sub-Saharan Africa (SSA) is specifically challenged with an average maternal mortality rate of 533 maternal deaths per 100,000 live births, newborn mortality of 42.7 per 1000 live births, and under-5 child mortality of 74 child deaths per 1000 live births [1]. According to the World Health Organization (WHO), pregnant women who do not receive adequate antenatal care have a ten-fold increased risk of mortality, while their infants have a three-fold increased risk of low birth weight and a five-fold increased risk of infant mortality [2]. While 85% of women in East Africa receive one antenatal visit, only 52% receive four antenatal visits, and this decreases to 47% in Rwanda [3]. Low utilization rates across SSA, including Rwanda, are often attributed to financial barriers, geographic distance, divergent cultural beliefs, lack of family support, and poor quality of care [4,5]. To reach the SDGs for maternal and child health, primary care delivery systems must understand and address these barriers to promote both early access and ongoing utilization of antenatal and pediatric services [5].

Routine antenatal care provided in a primary healthcare setting can prevent the morbidity and mortality associated with pregnancy and early childhood development [6]. Antenatal care includes preventative interventions, screening and treatment of potential complications, and health education to support a healthy pregnancy and safe childbirth [5]. Antenatal care should also enable women to take an active role in their care. Hope, with the psychometric factors of interconnectedness, readiness for change, and future orientation [7], is an essential precursor to this active engagement.

Hope plays a critical role in effective healthcare delivery by facilitating strong reciprocal relationships between healthcare providers (HCPs) and healthcare recipients (HCRs) [8]. Health systems that prioritize interconnectedness between HCPs and HCRs can strengthen the readiness and commitment of both populations to invest in positive future outcomes [9]. When systems fail to foster hope, HCPs experience burnout, leading to a decline in healthcare quality. Consequently, women tend to avoid seeking health care, resulting in adverse health outcomes [10].

The three psychometrics factors of hope are known to contribute to effective healthcare delivery. Interconnectedness, or the relationship between healthcare recipients and their healthcare providers, affects both engagement [11] and the caliber of care that HCPs provide [12]. It increases job satisfaction [13] and reduces burnout [14] among HCPs. Readiness for change, described as the “intersection of willingness to implement the desired change and the perceived ability to implement the change effectively” [15], is an influential component of adopting healthy behavior among HCRs [15], and implementing change among HCPs [16]. Future orientation refers to an individual’s beliefs and behaviors regarding their ability to improve their future circumstances. This includes anticipating feeling better in the future, making constructive plans, and achieving desired goals [17]. Future orientation has been linked to better mental and physical health, as well as improved health-related quality of life among individuals receiving care in primary healthcare settings [18].

HCPs are uniquely positioned to influence hope as they are engaged across various, often life-changing, stages of HCRs’ healthcare journey. The literature suggests they can either enhance, sustain, or diminish hope in HCRs through their attitudes, behaviors, and communication approaches [19]. While HCPs serve as critical sources of hope among HCRs, they are themselves suffering from rampant levels of burnout [20]. Burnout is characterized by depersonalization, emotional exhaustion, and a sense of professional failure [21]. It is known to cause poor quality care, further discouraging HCRs engagement and investment in care [21]. Consequently, HCPs are less likely to provide care that inspires hope and engagement among HCRs. While the role of hope in mitigating burnout among HCPs has been explored, few studies have investigated its role and influence on their capability, opportunity, and motivation to provide care that has the potential to inspire hope among the HCRs they care for [22].

Accordingly, this study aims to understand facilitators and barriers to hopefulness among HCPs and HCRs, specifically pregnant women (PW) and mothers of children under five years (MCU5), in Rwanda. This qualitative analysis will contribute critical insight for health system strengthening by identifying high-priority interventions to inspire hope among HCPs to provide high-quality care and among HCRs to actively engage in care and achieve SDGs for maternal and child health in Rwanda.

## Methods

### Setting

This study was conducted in rural communities within the catchment areas of three district hospitals (DHs) in Rwanda’s Northern Province. In Rwanda, each DH typically serves a population of approximately 200,000 people [23]. The first study site was Ruli DH, where TIP-Global Health (TIP-GH) [24], an international NGO, has maintained a presence since 2012. The second and third sites, Nemba and Kinihira DHs, were selected as locations where TIP-GH had no prior engagement before this study. The health facilities (HFs) within these catchment areas varied by classification (faith-based or public), geographic accessibility (including mountainous terrain and roads prone to flooding), and proximity to essential infrastructure. The number of HF partners supporting maternal and child health services was also considered during site selection.

### Design

We conducted a qualitative study using an Interpretive Description (ID) approach to examine how HCPs and HCRs perceive and experience hope within primary healthcare settings. To generate context-specific and practical insights, we used the ID approach to explore the individual, interpersonal, and systemic influences on hope to inform policymakers and practitioners. We used focus group discussions (FGDs) and key informant interviews (KIIs) as our primary data collection methods. We followed the Consolidated Criteria for Reporting Qualitative Research (COREQ) checklist to ensure transparency in reporting [25].

### Sampling

We aligned our sample size with established guidance on qualitative sample sizes necessary to achieve saturation and information power [26]. We used purposive sampling to recruit study participants, including HCPs involved in maternal and child healthcare services and HCRs, specifically pregnant women (PW) and mothers of children under five (MCU5). Given that the primary objective of this study was to examine general influences on hope within a primary healthcare context, women with diagnosed mental health conditions or critical illnesses were excluded. This exclusion was intended to minimize the impact of clinical factors that could substantially bias participants’ responses. Prior research indicates that individuals experiencing severe physical illness or psychological disorders may conceptualize and articulate hope in ways that differ markedly from those in stable health [7,27].

### Data collection tool

We conducted FGDs and KII using semi-structured, open-ended questions. To ensure that our data collection tools were meaningful and contextually appropriate, we drew on multiple theoretical frameworks, recognizing that hope is influenced by emotional, social, behavioral, and structural factors. This approach enabled us to capture the complexity and intersectionality of influences on hope among HCPs and HCRs. We began with the Kinyarwanda version of the Herth Hope Index (HHI-K) [28], a validated tool in Rwanda, to guide the structure and content of our interview and FGD guides. The HHI conceptualizes hope as multidimensional, comprising a sense of future and purpose (temporality), the belief that positive outcomes are possible (positive readiness), and the importance of relationships (interconnectedness).

For the HCP guide, we applied the Capability, Opportunity, Motivation–Behavior (COM-B) model [29] to examine factors that enable or hinder the delivery of hopeful and patient-centered care. The COM-B model considers how internal factors (e.g., knowledge, attitudes) and external factors (e.g., workplace environment, system-level supports) influence behavior, helping us frame questions to understand how HCPs enact hope-related practices in care delivery. For the HCR guide, we used the Health Belief Model (HBM) [30] to explore how individuals perceive health, what motivates them to seek care, and what fosters hope and engagement. The HBM informed questions on perceived risks, benefits of treatment, self-efficacy, and the social and emotional factors influencing care-seeking behaviors. Additionally, we applied the Socioecological Model (SEM) [31] to examine how individual experiences of hope are shaped by interpersonal relationships, community norms, and healthcare system structures, allowing us to consider contextual an environmental influences beyond individual-level factors.

We developed and refined all data collection tools through collaborative discussions within the research team. The final guides were translated into Kinyarwanda by a bilingual TIP-GH staff member.

### Data collection procedures

We collected data after obtaining verbal and written informed consent from all participants. We conducted 28 FGDs with 144 HCRs and 81 HCPs. Additionally, we conducted 23 KIIs with four policymakers, five researchers, and 14 health facility (HF) managers. All FGDs were held in healthcare facilities, while KIIs took place in participants’ local offices. Data collection occurred between 5 October 2020 and 10 February 2021. All sessions were conducted in Kinyarwanda and were audio recorded. FGD sessions lasted an average of 90 minutes, while KII sessions averaged 50 minutes. During data collection, the research team held monthly meetings to review interviewer field experiences and assess data saturation. As remuneration, FGD participants received 5 kg of Aheza, a locally produced fortified porridge.

### Data analysis

We conducted a thematic analysis guided by the definition of hope in three dimensions as indicated by the HHI-K and the SEM to explore both personal and contextual dimensions of hope. The HHI-K informed the coding of individual experiences by highlighting three dimensions of hope: future orientation, readiness for change and interconnectedness. The SEM complemented this by situating hope within broader social and structural contexts, allowing us to examine influences at the individual, interpersonal, community, and health system levels, and to understand how external conditions shaped participants’ ability to sustain hope.

We present emerging themes with attention to both shared and distinct experiences between HCPs and HCRs. Our thematic analysis followed seven steps to ensure rigor: (1) transcription; (2) familiarization through open reading; (3) coding; (4) developing a working analytical framework; (5) applying the framework; (6) charting data into a framework matrix; and (7) interpreting the data.

All audio recordings were transcribed verbatim in Kinyarwanda and translated into English by two independent transcribers to minimize bias. We randomly selected 28 transcripts for open reading and collaboratively developed an initial codebook with 20 codes through discussion among five researchers. This iterative process allowed us to refine and finalize the codebook. We uploaded all transcripts into Dedoose software (version 9.0.90) for systematic coding. Identified codes were organized into categories, which were further refined into themes and subthemes based on team discussions.

### Ethical consideration

We obtained ethical approval for this study from the Rwanda National Ethics Committee on 15 September 2020 (IRB 00001497, approval No. 692/RNEC/2020). We informed participants about the study objectives, assured them of voluntary participation, and guaranteed anonymity. Consent forms were read aloud in Kinyarwanda, allowing participants to ask questions before providing written consent. To protect privacy and confidentiality, we used personal identifiers only for initial contact, after which each participant was assigned a unique number stored separately from the data. All documents were securely stored on a password-protected laptop accessible only to the research team. During analysis, presentation, and publication, we used only unique identifiers. To safeguard participants’ mental well-being, we designed questions to minimize the risk of distress and trained data collectors to provide referrals for support if participants expressed psychological distress during data collection.

## Findings

### Participation and participants’ characteristics

Of the 28 FGDs conducted, among HCPs (n = 81), 63% were female, while nearly all HCRs (n = 144) were female (99%). HCPs were generally older, with an average age range of 37 years compared to 29 years for HCRs. Most HCPs had higher education levels, notably advanced diplomas (51%), whereas the majority of HCRs had primary education (74%). Regarding marital status, most participants in both groups were legally married. Table 1 presents the demographic characteristics of HCPs and HCRs who participated in the study.

**Table 1:** Demographic information for focus group participants.

Characteristics	Response	HCPs N (%)	HCRs N (%)
**Gender**	Female	51(63%)	143(99%)
	Male	30(37%)	1(1%)
**Age**	Min	23	18
	Max	65	48
	Average	33	37
	18-29	18(22.5%)	73(51%)
	30-39	36(45%)	57(40%)
	40-49	17(21.25%)	14(10%)
	50+	9(11.25%)	0%
**Level of education**	None	0(0%)	13(9%)
	Primary	3(4%)	107(74%)
	Vocation	3(4%)	4(3%)
	Secondary school	22(27%)	17(12%)
	Advanced Diploma	41(51%)	1(1%)
	Bachelor degree	11(14.%)	2(1%)
	Masters	0%	0%
**Ubudehe Category*(Social economical categories)***	Cat 1	1(1%)	17(12%)
	Cat 2	12(15%)	66 (46%)
	Cat 3	66(83%)	61 (42%)
	Cat 4	0 (0%)	0 (0%)
**Marital status**	Single	21(26%)	33(23%)
	Legally married	54(68%)	86(59%)
	Living with a partner	0(0%)	24(17%)
	Religious	1(1%)	1(1%)
	Religious Divorced	3(4%)	0%

Of the 23 individuals participated in the KII, 58% of them were male and most were aged between 31 and 50 years (89%). Participants were generally well-educated, with 42% holding a bachelor’s degree and 37% having attained a master’s degree or higher. Almost all participants (95%) reported being legally married. Table 2 summarizes the demographic characteristics of the KII participants.

**Table 2:** Demographic information for Key Informant Interview participants.

Characteristics	Response	N (%)
Gender	Male	13(58.%)
	Female	10(42%)
Age	31-40	10(42%)
	41-50	11(47%)
	50+	3(11%)
Education	Advanced Diploma (A1)	5(21%)
	Bachelor’s Bachelors’ degree (A0)	10(42%)
	Master’s degree and higher education	9(37%)
Marital status	Single	1(5%)
	Legally married	22(95%)

### Thematic analysis and factors influencing hope in healthcare settings

The analysis highlighted how personal, interpersonal, and system-level factors shape hope of HCPs and HCRs. For HCRs, individual and interpersonal factors acted as both facilitators and barriers to hope. For HCPs, these factors primarily facilitated hope, while systemic factors presented the main barriers. Four main themes emerged from the analysis. S1 and S2 Files provide detailed subthemes and definitions, organized under the three psychometric dimensions of hope, for themes related to HCPs and HCRs, respectively.

Theme 1: Relationships driven by HCPs influence the quality of care.

Theme 2: Perceptions of the health system are formed through direct encounters with care delivery.

Theme 3: Continuous learning, knowledge sharing, and religious beliefs are fundamental facets for readiness for change and future orientation.

Theme 4: Interplay of HCPs’ passion for their role, work environment, and resource availability in shaping quality of Care.

### Theme 1: Relationships driven by HCPs influence the quality of care

#### HCPs’ Compassionate communication.

Respondents highlighted the pivotal role that friendly and effective communication plays in fostering positive connections between HCRs and HCPs. HCPs noted that establishing a connection with HCRs is crucial in building trust, leading to the provision of high-quality services and a positive overall experience for both parties. HF leaders further emphasized the importance of HCPs’ demonstration of compassion and kindness in their communication with HCRs to foster a sense of interconnection.

*“Welcoming a client, whether a local community member or a patient, means showing them that you are with them and that you care. If you do not show that you care, they will in turn, feel that indifference. Normally, as a HCP, you need to understand their concerns and put yourself in their shoes. When you treat their concerns as if they were your own, they open up to you more. As a result, they are more likely to adhere well to healthcare services”. HCP-14*

Additionally, line managers highlighted the necessity for ongoing capacity building among HCPs to enhance their skills in engaging with and relating to HCRs. One line manager LM-9, mentioned performing training sessions with HCPs to discuss ways to establish a relationship with HCRs and provide a sense that they are part of a family and that they will receive quality services.

*“But again, an employee who is not capable... actually, you connect with a person because they trust you. Sometimes, when a client comes in and realizes that the person serving them is providing unclear or inconsistent information, it causes them to lose trust. As a result, the sense of engagement diminishes”.* *LM-14*

#### Mutual support and mutual respect.

HCP described how feeling supported by colleagues and treated with respect by peers contributed to a more cohesive and trusting workplace. This interpersonal harmony improves morale and enhances the HCPs’ sense of purpose and capacity to offer empathetic care. Healthcare administrators mentioned the importance of relationships between HCPs in the workplace, underscoring their direct impact on interactions with HCRs. They highlighted that a positive workplace experience is instrumental in enhancing problem-solving abilities and elevating the quality care delivery. LM-13 adds that the way HCPs interconnect is a powerful pillar that keeps their work running smoothly because they co-create a supportive and trust-based environment where they can rely on one another and make their service delivery successful.

*“Let me start with the interaction between me and a patient seeking care. The way I behave when they approach me will influence how open they feel with me. If I act like a high-level healthcare provider, they might immediately think, ‘What can I possibly say to someone who thinks they’re above everyone?’ The same applies with colleagues. If I act like I know everything and dismiss their contributions, we lose the opportunity to collaborate and communicate effectively. When communication breaks down, colleagues may think, ‘Even if I do this well, they will just criticize me’. Do you see how working in a team where someone acts superior can create disconnection? Even when you need help, others may withhold support, believing you will never be satisfied with what they offer because you already see yourself as above them.” HCP-7*

*Another way we try to support our HCPs, even though it can be challenging, is by working as a team to prevent anyone from becoming fatigued and losing that connection among staff members. As a leader, you observe how different services are functioning. If you notice an overloaded HCP and see another who isn’t, you ask the latter to support the one who is overwhelmed. This helps prevent fatigue among HCPs and ultimately reduces the time spent per client. LM_14*

### Theme 2: Perceptions of the health system are formed through direct encounters with care delivery

#### Positive interactions with HCPs.

HCRs expressed that when received with a friendly demeanor at the HF, it enhanced their comfort and ease with the HCPs. This emotional safety, in turn, encouraged them to open up, ask questions, and participate more actively in their care. HCR-5 indicated that her experiences with HCPs who provide care for her children’s health influence not only hope for herself but also her community. “I do not keep that hope to myself; I share it in my family, among mothers in my neighborhood, and even with mothers who neglected their responsibilities toward their children.” (HCR-5)

*“We are all created differently... You [HCR] might arrive at the health center, and even before sitting down, a HCP approaches you and asks, ‘You came to the HC?, Have you come to get your child treated?’ Then you begin to explain. That person who showed kindness from the beginning, next time, you will do your best to find that same HCP again, seeking their advice on where to go, how to get there, and what to do, depending on the service you need. ”. HCR-1*

#### Health facility reputation.

HCRs stated that their trust in the health system’s ability to meet their future health needs is influenced by prior interactions with the health system, including their perception of the care they or their family members have received at the health facility. While positive interactions with the health system lead to favorable health outcomes, negative interactions cause HCRs to be dissatisfied with healthcare delivery. HCPs indicated that displaying poor behavior towards HCRs could undermine their willingness to be transparent when sharing information, eventually affecting the quality of care received.

*“I can have hope for the future because of the way I am being treated. I was once a caregiver [someone who stays with a sick person, especially in public health centers in Rwanda], and the person I was caring for recovered because of the quality healthcare services they received. That experience strengthened my belief that whether I am sick myself or acting as a caregiver again, I will go to the health facility because I trust that I will receive quality care. I believe I, or the person I’m caring for, will recover because we are well taken care of.” HCR-6*

*You can refuse to accept a change, depending on what it is. For example, you might come for treatment with a specific concern, and because you already know that this health facility doesn’t have the equipment or advanced knowledge to manage your case, you request a referral [the HC gives you a referral paper that allows you to accessing the district hospital services]. But instead, they refuse and accuse you of being disrespectful, saying, ‘If that’s how you feel, you can treat yourself.’ In that case, do you think you would go back to that health facility? HCR-4*

#### System infrastructure.

HCRs are also critically affected by the limited availability of resources and finances in the healthcare system, which facilitates access to tertiary healthcare services and ensures continuity of care. Affected cases are illustrated by the inability to reach the hospital on time and receive urgent support due to a lack of transportation provided by the healthcare system (ambulances), which is met with an expensive cost of transportation, an expensive cost of hospitalization, confusion in filling out medical documents, and a shortage in the number of HCPs at the healthcare facilities.

*“I gave birth to a child who has an eye disability. They [HCPs] told me to take the child to [name of the hospital], and as someone who is in the third category of ubudehe [middle-class individual in the Rwandan socio-economic classification system, who is not eligible to receive government support], how can I go to [name of the hospital] while I know that they will refer me [my child] to the higher hospital? What will I do with insufficient means? So, I chose to stop going there because of my limited financial capacity. HCR-10.*

### Theme 3: Continuous learning, knowledge sharing and religious beliefs are fundamental facets for readiness for change and future orientation

#### Developing a shared vision of health goals with HCRs.

HCRs noted that the provision of health education by HCPs, clear communication, and engagement that concerns their health conditions enhances their readiness for change. HCR-4 provided the example of when HCPs prescribe pain medication without explaining their source of pain. “It would be better if they [HCPs] would explain to them [HCRs] to the extent that they can understand and be aware of their illness and how to protect themselves,” they add.

*“For me to accept change without any difficulties, the HCPs can frequently establish a program of teaching us [mothers], and local leaders can do the same, not only here at the HF but also in meetings such as parental evenings. They [local leaders] can discuss with us, and we can participate in government activities and programs instead of always being left behind at home.” HCR-9*

Respondents stated that there were several factors that could undermine future orientation in the healthcare system. HCRs mentioned that perceived ineffective treatment was a major barrier to developing a future mindset or vision, as well as personal matters in their household. Moreover, health system leaders and managers explained that HCRs struggle to accept change when it does not yield the expected outcomes. In addition, HCPs highlighted the need to explain changes to HCRs to support their readiness for implementation. Both leaders and HCPs underscored the value of educating HCRs about their rights and responsibilities, as well as the roles and responsibilities of the HCPs who care for them.

#### HCPs capacity building for change.

HCPs described capacity building through training, peer learning, and access to supportive tools strengthened HCPs’ capacity to adapt to change. Healthcare leaders emphasized the importance of informing HCPs about the rights of HCRs and effective approaches for communicating with patients experiencing pain or distress.

*The fact that you can see change and accept it quickly shows that you have been prepared for that change. It shouldn’t be things that suddenly happen to you and then you have to adapt. Let’s return to the example of electronic billing. If such a change occurs and they give us at least 15 or 30 days’ notice, then we would be ready when the system is implemented because we would have been trained on how to use it. It is easier if we are prepared in advance rather than having change fall on us unexpectedly like rain; in that case, it becomes difficult to get ready for sudden change. Yes, you accept it because there is no choice, but a change that someone is unprepared for is hard to get used to. HCP-3*

On the other hand, HCPs expressed that unofficial sources of information and inconsistent guidance about changes in service provision were obstacles to their readiness to change. This in turn affects their communication with HCRs through inadequate provision of information, which increases their struggle to accept change.

#### Mindset and religious beliefs.

Many HCRs also mentioned that religious faith helps make it easier to accept change. “Things that make it easier for us to accept changes! When you encounter those changes, you kneel and pray. As you have prayed to God, he helps you to accept changes,” says HCR-10. Religion was also noted to affect the readiness for change for HCPs, as they viewed it as a personal factor that affected their willingness to provide care. HCPs noted some situations, including provision of contraceptives, abortions, insertion of DIU (Intrauterine Device), family planning, and others, where resistance to receiving or providing such care could be due to religious beliefs, resulting in poor service provision and a decreased sense of hope. Additionally, LM-1 noted that mindset sometimes makes it difficult for HCRs to have a healthcare vision, in contrast to others who are responsive to the IEC [Information Education Communication] given by HCPs.

*“Those who have a lot of kids tell you “God will raise them” when you are teaching and asking, “Why don’t you consider family planning?” They respond, “God will take care of them.” You can see that they do not have vision.” LM-1*

### Theme 4: Interplay of HCPs’ passion for their role, work environment, and resource availability in shaping quality of Care

#### Nurturing passion.

HCPs highlighted love and dedication as key to the HCP-HCR connection. Their career drive and care for HCRs also enhance this bond. They described how imbalance between HCRs rights and rights of HCPs affects their motivation and professional identity. Health leaders advocate aligning HCPs’ vision with the institution’s for future orientation. HCPs emphasize leaders’ roles in aligning personal goals with institutional vision.

*“But the system has made it difficult because of these client rights. I don’t know; they should revise that and find an alternative. I admit we’re not blameless, but it really feels dishonoring to us...We can be overloaded and fail to do everything, and when a mistake happens, they report us, and we have no where to explain. They only listen to the client’s side, and we end up being blamed... This approach of treating the client as always right discourages us nurses. They post announcements saying, ‘At this facility, mothers and infants are dying,’ but they do not listen to the nurse or ask for their opinion. That’s why young people turn away from midwifery, and those who can manage prefer nursing, even though both have risks.” HCP-13*

*“To have a vision starts within you. A person will not sit here to have a vision in a health domain without even having a vision in their life. So, when a person arrives at the HF and is determined to excel in their job, they will experience good leadership. A good leader will guide them well, give them space and time, and equip and train them so that they can have a career path and vision”. HCP-3.*

Moreover, HCPs emphasized the importance of recognition and rewards from leadership in driving their future orientation. This includes fair salary scales, bonuses, and promotions. When recognized and motivated, they provide higher-quality care. However, unclear work prospects and unsatisfactory conditions hinder their ability to set clear goals and action plans, impacting their future mindset. Challenges include inconsistent performance-based financing, inadequate salary schemes, and limited resources for equipment, affect HCPs’ performance.

*“Other things that could prevent them [HCPs] from having a vision... sometimes, salary can play a role in not having a vision. Because salaries go hand in hand with national resources. We don’t ignore it. but the salary is also affected by the economic changes happening. You find that the money earned can’t cater to their daily needs, such as caring for children and paying for education. These can affect them [HCPs], causing them to focus less on the patients since they are spending half of their attention and concentration on thinking about how to fix those things. They will look for other supplement work.” RES-5*

#### Workforce dynamics and capacity: workforce strain.

Healthcare facilities face human resource shortages. This increases workloads and working hours, leading to burnout and hindering future orientation for HCPs. Insufficient staff relative to services and HCRs affects care quality and future focus. Short consultation periods, severity of cases and time constraints impact care quality and hinder HCP-HCR connection. In addition, increased workload and burnout weaken team dynamics, affecting the connection among HCPs and their leaders, and ultimately affecting the quality of care for HCRs.

*“Now we have less staff [at the HF]. You find yourself covering at the same time the consultation service, pharmacy, and CPN services (Prenatal care services). Because of the number of tasks and our scarcity, it will not be easy for us to achieve our goal. The person works just to get patients out of your way [HCRs leave the HF], but sometimes we don’t provide quality care because of the number of departments to cover and people to receive. HCP-11*

## Discussion

This study explored how HCRs and HCPs experience and conceptualize hope within primary healthcare settings. Guided by the Herth Hope Index (HHI), the findings highlight factors that influence three core dimensions of hope: interconnectedness, readiness for change, and future orientation. These are shaped by personal experiences, systemic conditions, and relational dynamics at various levels of the health system.

### Interconnectedness

Participants consistently emphasized that human connection is foundational to care. HCRs valued respectful engagement and open dialogue, explaining that they were more likely to follow health advice when they felt genuinely seen and heard by their HCPs. These findings are echoed in prior studies linking HCPs-HCRs communication to satisfaction [32], better health outcomes [33], and service quality [34].

HCPs, in turn, expressed a sense of duty to build these relationships. They recognized that trust and rapport helped with care adherence and reducing skepticism at the community level. As others have found, strong interpersonal relationships can reinforce continuity of care and adherence to treatment [35,36] and even support psychosocial healing [37].

Connections among HCPs were equally important. Respondents described how peer support, teamwork, and open communication among colleagues and with leadership promoted a safer and more encouraging work environment, needed for delivering consistent care. Previous work highlights that peer collaboration enhances quality [38], supports patient safety [39], and fosters positive safety culture when backed by leadership [40–42].

HCPs identified systemic factors as barriers to building connections with HCRs and colleagues, which in turn diminished their sense of hope. Work overload, limited staff, and strained supervision led to burnout and occasionally disrespectful behaviors. Some HCPs noted that seeing their role as a “calling” rather than just a job helped them stay grounded and committed, even in difficult settings. However, as shown in other contexts, unresolved interprofessional conflicts and poor morale can erode the very relationships that uphold care quality [43,44]. Leaders also shared that misinformation, time constraints, and staff shortages impaired opportunities for meaningful interactions with HCRs as factors that ripple through the quality of care [45,46].

### Readiness for change

People’s willingness to engage with new information or change their behavior closely depended on how much they trusted the source and how clearly the message was communicated. HCRs described how clear health education sessions and respectful dialogue increased their openness to change. In contrast, when HCPs gave rushed or inconsistent explanations, HCRs became doubtful or resistant. These dynamics align with evidence showing that unclear communication undermines adherence [47], while accessible information helps people navigate systemic barriers [48].

Negative care experiences such as ineffective treatment eroded the willingness to accept change. As WHO and others have emphasized, the experience of care is as vital as its content [49]. Without a sense of being heard or respected, both trust and behavior change suffer [50,51].

Belief systems and religion were central to how change was processed. Many participants shared how faith helped them cope with uncertainty and guided decisions. However, in some cases, religious convictions created tension with health interventions, particularly around family planning, echoing findings from Zimbabwe and other Low and Middle-Income Countries (LMICs) [52–55].

Among HCPs, readiness for change was strengthened by regular communication, training, and participatory leadership. Many HCPs and leaders noted that inadequate explanation of new guidelines hampered adoption. Studies show that communication channels and clear training significantly influence the uptake of change [56,57]. Peer learning and on-the-job coaching were seen as effective in preparing HCPs to adopt new practices [55,58], while leaders saw their role as facilitators of change through consistent engagement and shared vision [59].

### Future orientation

Hope for the future perceived as both personal and systemic influences, was shaped by past experiences. HCRs described feeling more optimistic and confident in the health system when they received effective care, were treated respectfully, or witnessed recovery of a loved one. These experiences deepened their belief in the health system’s potential. Similar findings have been reported in maternal care, where quality and responsiveness influence satisfaction and use of services [41,60,61].

Conversely, poor outcomes particularly unresolved illness or ineffective treatment, left lasting impressions. “Not getting healed” was a common phrase, underlining how failed care can diminish hope not only for the individual but for their family and community. Word-of-mouth was a powerful force in shaping future expectations about care, especially in rural or tightly connected communities [62].

For HCPs, their outlook was influenced by workplace dynamics. Feeling appreciated, having a clear organizational vision, and receiving feedback were cited as key motivators. Prior studies have shown that these factors can strengthen creative performance and psychological resilience [63,64].

On the other hand, high workloads, resource scarcity, and inadequate pay undermined morale. Many HCPs expressed that their salaries did not meet basic living needs, which led to stress and a sense of stagnation. These realities mirror well-documented challenges in LMIC health systems [65–67], where staff shortages and low compensation fuel turnover, dissatisfaction, and burnout [68,69]. As echoed in the WHO framework, material and emotional support are both essential for sustaining professional engagement [59].

The findings of this study resonate with key constructs from the COM-B model, the Health Belief Model (HBM), and the Social Ecological Model (SEM), each offering a complementary lens through which to understand the dynamics of hope in primary healthcare. For example, participants’ sense of interconnectedness was reported to be shaped by respectful HCP-HCR interactions and peer support, reflects the SEM’s emphasis on interpersonal and organizational levels of influence on behavior [31]. At the same time, the readiness for change expressed by both HCRs and HCPs aligns with the COM-B components, suggesting that behavior change is rooted in multi-level enablers [29]. In this research, those enablers were highlighted with capability (through knowledge and training), opportunity (via supportive leadership), and motivation (driven by trust and belief in care). The Health belief model (HBM) was also evident in how HCRs described their likelihood of engaging with services, based on perceived benefits such as respectful treatment and perceived barriers such as poor communication or religious concerns. Cues to action, such as witnessing a peer’s recovery or receiving clear education is another component of HBM that was described by HCRs. These frameworks collectively illuminate how hope is constructed not just through individual beliefs but also within social and systemic environments, reinforcing the need for people-centered care strategies that address behavioral, relational, and structural factors.

In the Sustainable Development Goal (SDG) era, advancing maternal and primary healthcare requires more than service expansion, it requires investing in the human experience of care [67]. Hope, as this study shows, is not an abstract ideal. It is built or broken through every interaction, policy, and system decision. Recognizing the relational, behavioral, and structural influences that shape hope can inform more responsive, resilient, and people-centered health systems.

## Conclusion

Our findings reinforce the important role of hope- interconnectedness, readiness for change, and future orientation- in delivering effective primary health care in Rwanda. According to WHO, the experience of care is an essential dimension to defining quality of care [56]. Definitions of quality care must be informed by the factors influencing hope among HCRs to encourage ongoing participation in care. To effectively strengthen primary health systems, governments and health system leaders must establish an enabling and hopeful environment for HCPs to provide this level of high-quality care and achieve the desired improvements in maternal and child health outcomes.

### Recommendations

Positive relationships between HCPs and HCRs must be prioritized in Rwanda’s primary healthcare facilities. This requires implementing practices that promote clinical best practices, patient-centered care, effective communication, and trust-building to deliver high-quality healthcare services. Recognizing the profound impact of personal beliefs and attitudes on hope, capability building and knowledge sharing on these factors are crucial. This enables the HCPs to address biases and prejudices that may affect the delivery or reception of quality maternal health services.

By investing in the development of positive relationships, awareness, and education, the primary health system in Rwanda can create a more hopeful and supportive environment for both HCPs and HCRs, ultimately leading to improved healthcare outcomes and experiences.

Regular monitoring, evaluation, and assessment of HCR’s experiences in health care are essential to gathering first-hand valuable insights on the effectiveness of healthcare quality. Additionally, regular monitoring of the support that HCPs receive from their leaders fosters a positive and productive work environment.

Given resource shortages in Rwanda, coordination and collaboration among all stakeholders involved in maternal health should prioritize resource availability and overall service quality. Interconnectedness, readiness for change, and future orientation are essential elements for delivering effective primary health care that achieves desired improvements in maternal and child health outcomes in Rwanda.

## Supporting information

S1 File This file contains definition of subthemes related to HCRs’ influencers of hope. (DOCX)

S2 File This file contains definition of subthemes related to HCPs’ influencers of hope. (DOCX)
