# Supplementary material for: Nurturing hope in Rwandan healthcare settings: Exploring factors that influence hope among healthcare providers, pregnant women, and mothers with children under five years
Source: PLOS Glob Public Health. 2025 Aug 29;5(8):e0005095. doi: 10.1371/journal.pgph.0005095 (PMC12396647; doi:10.1371/journal.pgph.0005095)
Supplement: S1 File — (DOCX) [file pgph.0005095.s001.docx]

**Healthcare Recipient Sub-themes and Definitions**

| **Interconnectedness** | |
| --- | --- |
| **Sub-Theme** | **Definition** |
| Trust | Capacity and comfort with open communication |
|  | Patient experience impacts future trust (ex. Broken confidentiality) |
|  | Effective communication by HealthCare Providers (HCPs) builds trust for HealthCare Recipients (HCRs) |
|  | HCW recognition of HCR’s life experience and conditions build trust |
|  | Correct/effective care provided builds trust |
|  | Access to feedback (health outcome improvements) |
|  | Ongoing care enhances patient trust and engagement |
| Mutual Respect | Welcoming and conducive environment to receive care |
|  | Shared vision of health goals |
|  | Ability to overcome intimidation from perceived/actual power dynamics |
| Social Cohesion/Inclusion | Learning through shared experience |
|  | Socioeconomic factors (Stigma related to poverty) |
|  | Personal bonds strengthen community ties among HCRs |
| **Readiness for Change** | |
| Trust | HCP communication/teaching style – describe benefit of change & facilitate problem-solving to overcome barriers. |
|  | Trust in health system |
| Social Inclusion/Cohesion | Social capital (seen as a visionary/leader) |
|  | Change through connection: Friends, colleagues, and family shape behavior |
| Mindset/Intrinsic Factors | Financial security: Financial capacity in enabling individual behavior change |
|  | Fair distribution of social support services (health insurance, Nutritional support,..) |
|  | Self-efficacy |
|  | Spirituality |
|  | Access to trauma-informed care |
|  | Capacity for change management |
| Learning/Knowledge | Cultural norms’ influence on capacity to act on knowledge |
|  | Training Style |
|  | Opportunities for engagement in development of intervention |
|  | Access to ongoing channel of communication/training and support |
| Clear Rights & Responsibilities | Awareness of HCRs’Rights and Responsibilities |
| **Future Orientation** | |
| Trust | Access to quality care at the right time and place |
|  | Access to feedback/progress updates |
| Mutual Support | Peer goal-setting and support |
|  | Family Support |
| Mindset/Intrinsic Factors | Ability to set goals and create/execute on plan to achieve them |
|  | Prior experiences/trauma influence ability to set and act upon future goals. |
| Resource Availability | Access to Resources/External Support |
|  | Financial Security |
