# Supplementary material for: Nurturing hope in Rwandan healthcare settings: Exploring factors that influence hope among healthcare providers, pregnant women, and mothers with children under five years
Source: PLOS Glob Public Health. 2025 Aug 29;5(8):e0005095. doi: 10.1371/journal.pgph.0005095 (PMC12396647; doi:10.1371/journal.pgph.0005095)
Supplement: S2 File — (DOCX) [file pgph.0005095.s002.docx]

**Healthcare Providers Sub-Themes and Definition**

| **Interconnectedness** | |
| --- | --- |
| **Sub-Theme** | **Definition** |
| Trust | Building trust and relationships as a core part of Health Care Providers' (HCPs) job |
|  | Respectful attention to Health Care Recipients (HCRs) enhances trust and reputation |
|  | Access to feedback mechanisms |
|  | Trust in self/self-efficacy |
|  | Patient experience impacts future trust |
| Passion for Role | HCW-HCR relationship is uniquely meaningful |
|  | Continued patient interactions reinforce HCPs’ sense of purpose. |
|  | Emotional investment in people and outcomes |
|  | Role as a vocation vs job |
|  | “Soft Skills” valued as of equal importance as Technical Skills |
| Leadership | Organizational culture that facilitates & maintains HCPs-HCRs relationships |
|  | Leadership style: Collegial support fuels accountability and performance. |
|  | Clear HCPs’ Rights & Responsibilities |
|  | The impact of insufficient support in managing challenging client behavior on HCP’sMorale |
| Mutual Respect | Openness to develop mutual respect between HCPs & HCPs & management |
|  | Access to consistent space and time for collective problem-solving |
|  | Opportunities to build camaraderie among HCPs |
|  | Sense of belonging drives initiative |
|  | Skills to manage HCR opposing views of treatment |
| Social Inclusion | Supportive personal relationships boost healthcare workers’ performance |
|  | Personal life influences professional perspective |
|  | Peer support |
| System & Infrastructure | Financial support (salaries) |
|  | Availability of medical equipment |
|  | Consistent space and time for collective problem-solving |
|  | Systems that support ongoing connections, deepen impact. |
|  | Clarity of HCW rights and responsibilities, supported by leadership, enhances performance and accountability. |
| **Readiness for Change** | |
| Trust | Trust in science and evolving evidence-based policies |
|  | Trust that the requested change will lead to change for the better |
|  | Leadership-Staff relationships as a catalyst on HCP’s Behavior Change and trust |
| Leadership | Leadership style that values and respects employees |
|  | Leadership provides support through the change that mitigates risk |
|  | Recognizes and celebrates staff accomplishments |
|  | Teamwork and information exchange drivers of HCW change |
| System & Infrastructure | Adequate equipment and procurement strategies |
|  | Effective and consistent training, staff, and materials |
|  | Structure and integration of complementary systems (health insurance, nutritional support, etc.) |
|  | System infrastructure ‘normalizes’ change/quality improvement processes |
|  | Consistent guidance, monitoring, and feedback supports change |
|  | System provides space/time to accommodate change (i.e. Work/patient loads) |
| Learning & Knowledge | Peer exchange fosters learning and adoption of change |
|  | Evidence-based explanation of benefits of change delivered in a way that promotes understanding/comprehension. |
|  | Learning and sharing best practices is an incentive to change |
|  | HCR health illiteracy/misinformation |
| Intrinsic Factors | Ability to accept and process feedback |
|  | Consistency with personal beliefs and requested change |
|  | Flexible/inclusive approach to introducing change based upon personal/individual factors. |
| **Future Orientation** | |
| Trust | Inclusive goal-setting (organization engages HCPs in process) |
| Leadership | Leadership style that supports and encourages professional growth |
|  | Being valued/recognized inspires a visionary mindset |
| System & Infrastructure | A system that creates an environment where people can recharge, take breaks, and think creatively, making it easier to manage workloads and invest in a better future. |
|  | Adequate equipment to perform current work enables the ability to consider future goals. |
|  | Work environment that provides opportunity and support for professional advancement |
|  | Access to financial resources for investment (cooperatives, savings, etc.) |
| Learning & Knowledge | Skillsets and training to support achievable goal setting |
| Passion for Role | Financial security enhances HCPs' visionary mindset and personal fulfillment. |
|  | Celebration of successes fuels passion and makes future goals feasible |
|  | Time to rejuvenate |
| Mutual Respect | Mutual inspiration fuels HCPs’ personal and professional success |
| Mindset/Intrinsic Factors | Personal insight, capacity for future-oriented mindset: Purposeful living as a source of intrinsic motivation and career commitment among HCPs facing chronic challenges. |
